# Evaluating High and Low Heart Rate Variability Response and Neurocognitive Performance in Workers: An Exploratory Study

**DOI:** 10.3390/bs13090742

**Published:** 2023-09-06

**Authors:** Ardalan Eslami, Najah Therese Nassif, Sara Lal

**Affiliations:** 1 Medical Innovation Neuroscience Data-Analytics (MIND) Unit, TD School, University of Technology Sydney, Sydney 2007, Australia; ardalan.eslami@uts.edu.au; 2 School of Life Sciences, University of Technology Sydney, Broadway, Sydney 2007, Australia; 3 School of Psychology, Faculty of Science, University of New South Wales, Sydney 2052, Australia; 4 School of Public Health, University of Technology Sydney, Broadway, Sydney 2007, Australia

**Keywords:** heart rate variability, high HRV, low HRV, cognitive function, autonomic nervous system, working memory, attention

## Abstract

Heart rate variability (HRV) has the potential to be a predicting factor of cognitive performance. The present research aimed to explore the differences in neurocognitive performance of workers with high HRV and low HRV. A total of 48 white-collar workers and 53 blue-collar workers were assessed. An electrocardiogram was used to obtain HRV data, whereby a 10 min baseline and an active (neuropsychological task) recording were taken. Median splits were performed on data to obtain high- and low-HRV groups. The Cambridge Neuropsychological Test Automated Battery, specifically, the spatial working memory, attention-switching task, rapid visual processing, and spatial span were used. Higher HRV (RMSSD and HF) was linked to better neurocognitive performance measures. Interestingly, the blue- and white-collar groups exhibited different correlations and, in some cases, showed an inverse relationship with the same variables. The differences observed in the present study demonstrate the importance of assessing task-dependent HRV parameters.

## 1. Introduction

Executive function pertains to a group of mental processes utilized for concentration and attention [1,2]. The utilization of executive functions entails considerable cognitive demands. Specifically, individuals tend to find it more convenient to persist with established habits rather than making changes, and they often default to an “autopilot” mode, where actions occur effortlessly without conscious deliberation about what to do next. Cognitive functioning gives rise to various derivations such as problem solving, planning, reasoning, multi-tasking, sustaining attention, and decision making [3,4]. In addition, these higher-order cognitive abilities are essential for mental and physical health, which have been extensively covered throughout the literature [5,6,7].

Key components of neurocognitive performance include working memory (WM) and attention. Working memory refers to an array of systems that are assumed to be necessary for short-term information storage as well as the manipulation of that information [1,8,9]. Inhibitory control involves the ability to control attention, behavior, emotions, etc., to over-rule an internal predisposition and, thus, do what is appropriate [1]. The inhibitory control of attention allows us to attend and focus selectively whilst suppressing attention to other stimuli. In addition, selective attention and WM are similar in various ways, including at the neural level, whereby the prefrontal cortex and posterior parietal cortex are crucial neural substrates for WM and attention [10].

Heart rate variability (HRV) has been extensively used to reflect the sympathetic and parasympathetic activity of the autonomic nervous system [11]. HRV has also been linked to various psychological processes [12,13,14]. These links prompt the question: can HRV be used as an independent factor in the prediction of cognitive performance [12]?

Low HRV has been linked to a low degree of neurovisceral integration and a reduced ability to assemble resources to meet the demands that are required in an attentional task [15]. Furthermore, vagal-mediated cardiac control has been shown to be pivotal in controlling attention, emotion, and other physiological processes [15]. Previous research has predominantly focused on clinical populations with pre-existing conditions, such as anxiety, and therefore, the predictive relationship between HRV, WM, and attention remains to be established in a healthy population.

People with higher HRV have indicated superior performance compared to lower HRV due to the increased vagal modulation in cardiac control [12]. Furthermore, lower vagal tone has been associated with a lower ability to match cognitive ability to environmental demands [12]. This reinforces the argument that HRV may be able to differentiate between good and poor performance in executive function and thus serve as a predictive measure in determining any impairments in cognition.

Previous research indicates a link between decreased HRV and increased WM load and higher HRV in better performers [16]. This supports the notion that HRV during working memory function may qualitatively predict cognitive differences among individuals [16]. This also implies that executive performance and autonomic functions, such as that of HRV, may be adaptively regulated by an inter-related neural network. Therefore, HRV may provide an index of an individual’s ability to function effectively in a dynamic environment [16].

Thus, the present study aims to address the void in the literature by investigating executive function, particularly working memory and attention, in two major working populations (n = 48 white collar and n = 53 blue collar). The use of validated, accurate, and reliable measures, such as heart rate variability and executive function assessments, will allow for comparative analysis to be subsequently performed. This will further identify how the neurocognitive performance of people with high or low HRV is influenced.

## 2. Materials and Methods

### 2.1. Recruitment

A total of 101 participants were recruited from the local community, comprising 48 white-collar workers with an average age of 40 ± 11 years and 53 blue-collar workers with an average age of 24 ± 2.4 years. We define blue-collar occupations as those that involve manual labor with much higher physical demands than white-collar occupations, which we define as managerial or administrative-based work. Before the testing session, all participants were screened for any health issues or diseases that have known effects on HRV and were excluded. Furthermore, participants were instructed to refrain from consuming caffeine and nicotine for a minimum of 4 h, as well as avoid alcohol for at least 12 h. These measures have been known to affect physiological measurements [17,18,19]. Written and informed consent was obtained prior to the experimental protocol. The University of Technology Sydney Human Research Ethics Committee (HREC: 2014000110 and HREC ETH19-3676) approved this study.

### 2.2. Experimental Protocol

Participants remained seated for at least 5 min prior to three blood pressure (BP) recordings using an automated monitor (OMRON IA1B, Japan). Three blood pressure readings were obtained both before and after the study protocol with 2 min intervals between each measurement [20]. Participants were then asked to complete the General Health Questionnaire (GHQ60) [21]. A baseline electrocardiogram (ECG) was recorded for 10 min followed by an active ECG taken during the neurocognitive tasks. The ECG was obtained using a FlexComp Infiniti encoder (Thought Technology Ltd., Montreal, QC, Canada) and an ECG-Flex/Pro amplifier sensor (Thought Technology Ltd., Montreal, QC, Canada) connected to three electrode leads. BioGraph Infiniti software (T7900) (Thought Technology Ltd., Montreal, QC, Canada) was used to record and display the ECG wave. Prior to placement of the electrodes, the skin was cleaned using Liv-Wipe (Livingstone International Pty Ltd., Mascot, Australia) 70% alcohol swabs. Disposable electrodes were used in all cases (Ag/AgCl ECG electrodes (Red Dot ^TM^) 2239, USA). The electrodes were placed in an inverted triangle to allow for positive deflections corresponding to the P, Q, R, S, and T waves [22]. The ECG was sampled at 2048 samples per second [23].

#### 2.2.1. Neuropsychological Assessments

The Cambridge Neuropsychological Test Automated Battery (CANTAB) was utilized in this study [24]. Specifically, we used the spatial working memory (SWM) task, which requires the retention and manipulation of visuospatial information; the attention-switching task (AST), which measures a participant’s ability to shift attention between tasks and to ignore irrelevant information during interfering and distracting events; the rapid visual processing task (RVP), measuring sustained attention; and the spatial span (SSP) task, which assesses working memory capacity [24].

#### 2.2.2. Heart Rate Variability Processing

The ECG data were pre-processed to obtain time and frequency parameters of heart rate variability (HRV). The ECG data were imported into Kubios HRV software (Version 3.1, University of Kuopio, Kuopio, Finland). The R-waves were automatically detected by applying the built-in QRS detection algorithm [25]. Frequency bands obtained were low frequency (LF) (0.04–0.15 Hz), high frequency (HF) (0.15–0.4 Hz), total power HRV (TP), and the ratio of LF to HF (LF/HF). The inbuilt process within Kubios and smoothness priors method was used to correct for artifacts and ectopic beats in the raw ECG data [25,26]. Statistical analysis was performed on the natural log of the pre-processed data.

### 2.3. Statistical Analysis

Statistical analysis was performed using SPSS Version 22.0 (IBM Corp., 2013, New York, NY, USA) [27], with statistical significance reported at *p* < 0.05. The Mann–Whitney U test was used to establish significant differences between the high-HRV and low-HRV groups. High-HRV and low-HRV groups were determined via a median split [12], which was performed on RMSSD, HF, and LF/HF parameters of HRV. The partial Spearman’s rank-order correlation was used to determine significant associations between HRV groups and the neurocognitive performance measures. Bonferroni corrections were applied in all instances where two or more independent variables were correlated to one dependent variable, and to determine the most significant predictor of HRV with cognitive performance, linear regression analysis was applied. Age, BMI, and sex were accounted for in all statistical analyses as potential confounding variables.

## 3. Results

### 3.1. White-Collar Workers

Median splits were performed on RMSSD, HF, and LF/HF (sympathovagal balance) [12]. This allowed for a comparison of neurocognitive performance between groups with low HRV and high HRV. As shown below in Table 1, the white-collar group had a median of 6.69 ms for log RMSSD, 5.07 ms^2^ for log HF, and 1.49 for log LF/HF.

#### 3.1.1. Differences in Neurocognitive Performance between High and Low HRV (White Collar)

Mann–Whitney U tests compared the neurocognitive performance scores between the high-HRV groups and the low-HRV groups for white-collar workers (Table 2). It was identified that the high log RMSSD group made fewer errors in the SSP task as compared to the low log RMSSD group, respectively (1.08 ± 1.2, 2.08 ± 1.44).

Conversely, the high log HF (high = 5.17 ± 2.44, low = 3.5 ± 2.35, respectively) group made more errors during the “side” cue in the AST.

#### 3.1.2. Correlations between HRV and Neurocognitive Performance Measures in High and Low HRV (White Collar)

To determine which associations were significantly related to dependent and independent variables, correlations were performed. Spearman’s bivariate analyses were performed between the high-HRV group and neurocognitive performance and between the low-HRV group and neurocognitive performance. As previously mentioned, the HRV parameters that were split were log RMSSD (parasympathetic activity), log HF (parasympathetic activity), and log LF/HF (sympathovagal balance) (Table 3, Table 4 and Table 5, respectively).

##### Log RMSSD

With a median of 6.69 (ms), the high log RMSSD group (Table 3) showed positive correlations between log RMSSD and total errors during the AST (r = 0.41, *p* = 0.049). A positive relationship was also found in the RVP task between log SDNN and reaction time (r = 0.6, *p* = 0.04), log LF and reaction time (r = 0.8, *p* < 0.01), log HF and reaction time (r = 0.9, *p* 0.01), and log TP and reaction time (r = 0.54, *p* < 0.001). The high RMSSD group also showed a negative correlation in the AST between log LF/HF and reaction time (r = −0.42, *p* = 0.04).

The low log RMSSD group (Table 3) identified negative associations between log SDNN and reaction time (r = −0.43, *p* = 0.04), log TP and reaction time (−0.46, *p* = 0.02), and also during the SWM task between log HF and errors on the four-box trial (r = −0.41, *p* = 0.045).

##### Log High Frequency

With a median of 5.07 (ms^2^), the high log HF group (Table 4) indicated a positive relationship between log SDNN and congruent errors in the AST (r = 0.42, *p* = 0.045) and log LF/HF and errors made in the six-box trial during the SWM task (r = 0.47, *p* = 0.02). The high log HF group also identified negative associations between log LF/HF and reaction time on correct trials during the AST (r = −0.63, *p* < 0.01) and between log LF/HF and errors on the six-box trial of the SWM task (r = −0.47, *p* = 0.02).

The low log HF group (Table 4) showed a positive association between log SDNN and the total correct entries during the AST (r = 0.43, *p* = 0.04). Negative associations were identified during the AST between log LF and the total correct entries (r = −0.44, *p* = 0.03), log HF and congruent errors (r = −0.41, *p* = 0.046), log HF and reaction time when the “direction” cue was given (r = −0.41, *p* = 0.049), and log TP and total incorrect entries (r = 0.46, *p* = 0.03). A negative association was also found between log RMSSD and the errors made where a box was not shown in the sequence during the SSP task (r = −0.42, *p* = 0.04).

##### Log LF/HF Ratio

With a median of 1.49, the high log LF/HF group (Table 5) demonstrated a negative correlation during the SSP task between log RMSSD and errors where the box was not next in the sequence (r = −0.48, *p* = 0.02).

The low log LF/HF group (Table 5) identified positive associations during the AST between log LF and reaction time when the “direction” cue was given (r = 0.49, *p* = 0.01) and log TP and reaction time when the “direction” cue was given (r = 0.41, *p* = 0.049). Additionally, positive relationships were found during the RVP task between log SDNN and reaction time of correct trials (r = 0.42, *p* = 0.04), log HF and reaction time during correct trials (r = 0.51, *p* = 0.01), and during the SWM task between log LF/HF and errors in the six-box trial (r = 0.57, *p* < 0.01). The low log LF/HF group identified negative relationships between log LF/HF and reaction time during correct trials (r = −0.52, *p* = 0.01). During the RVP task, a negative association was found between log LF/HF and reaction time during the correct trials (r = −0.77, *p* < 0.001). Moreover, during the SWM task, negative relationships were found between log HF and errors during the six-box trial (r = −0.55, *p* = 0.01) and log TP and errors during the six-box trial (r = −0.44, *p* = 0.03).

A subsequent multiple regression was performed for the dependent variable log LF/HF (Table 6) using the significant correlations identified in Table 5 above (reaction time for correct trials during the AST and RVP task and errors made on the six-box trial of SWM). It was found to be overall significant for the white-collar LF/HF HRV (F(3,20) = 6.81, *p* = 0.002), and together the independent variables explain 43% of the variance in LF/HF of the white-collar (low) group. Reaction time for correct trials during the RVP task was the significant predictor (*p* = 0.008).

### 3.2. Blue-Collar Workers

Median splits were again performed for the blue-collar group (Table 7). The median splits separated the blue-collar group into high and low-HRV groups based on RMSSD, HF, and LF/HF.

#### 3.2.1. Differences in Neurocognitive Performance between High and Low HRV (Blue-Collar)

For the blue-collar workers, Mann–Whitney U tests were performed (Table 8) to identify any differences in neurocognitive performance between the high-HRV and low-HRV groups.

It was identified that the high log RMSSD group was worse at detecting sequences than the low log RMSSD group in the RVP task (0.8 ± 0.08, 0.9 ± 0.07, respectively). The high log RMSSD group detected fewer sequences (33.08 ± 12.26, 40.61 ± 9.79, respectively) and also missed more sequences than the low log RMSSD group in the RVP task (20.92 ± 12.26, 13.39 ± 9.79, respectively).

The high log HF group showed fewer errors during the SSP task as compared to the low log HF group (8 ± 5.95, 11.29 ± 6.56).

The high log LF/HF group was better at detecting sequences than the low log LF/HF group (41.19 ± 9.62, 33.07 ± 12.03) and also missed fewer sequences (12.81 ± 9.62, 20.92 ± 12.03) during the RVP task.

#### 3.2.2. Correlations between HRV and Neurocognitive Performance Measures in High and Low HRV (Blue Collar)

Spearman’s bivariate analyses were performed between high- and low-HRV groups (log RMSSD, log HF, log LF/HF) and neurocognitive performance (Table 9, Table 10 and Table 11, respectively).

##### Log RMSSD

The high log RMSSD group (Table 9) identified positive associations during the SSP task between log SDNN and the longest sequence measured (r = 0.49, *p* = 0.001), and log LF/HF and errors where the box was not in the sequence at all (r = 0.39, *p* = 0.04). Positive associations were also found during the SWM task between log HF and the longest sequence measured (r = 0.57, *p* = 0.002). Moreover, during the SSP task, negative associations were also identified between log RMSSD and the longest sequence measured (r = −0.4, *p* = 0.04), log SDNN errors that did not appear next in the sequence (r = −0.38, *p* = 0.049), and between log LF/HF and the longest sequence measured (r = −0.51, *p* = 0.002).

The low log RMSSD group (Table 9) identified positive correlations during the RVP task between log RMSSD and errors where the box was not next in the sequence (r = 0.44, *p* = 0.03). Positive correlations were also identified during the SSP task between log SDNN and errors where the box was not next in the sequence (r = 0.44, *p* = 0.03) and between log TP and reaction time on correct trials (r = −0.43, *p* = 0.03). Negative associations in the low log RMSSD group were found during the SSP task between log TP and the longest sequence (r = −0.4, *p* = 0.045).

##### Log High Frequency

The high log HF group (Table 10) showed positive relationships during the RVP task between log SDNN, log HF, log TP, and reaction time during correct trials (r = 0.54, *p* = 0.003; r = 0.41, *p* = 0.03; r = 0.48, *p* = 0.01; respectively).

The low log HF group (Table 10) indicated positive correlations during the AST between log SDNN, log HF, and the total incorrect actions (r = 0.54, *p* = 0.01; r = 0.41, *p* = 0.04; respectively). Additionally, a positive correlation was also found during the RVP task between log LF/HF and the total sequences missed (r = 0.45, *p* = 0.02). Negative associations were shown during the RVP task between LF/HF and total sequences detected (r = −0.45, *p* = 0.02). During the SSP task, a negative correlation was found between log SDNN and errors where the box was not next in the sequence (r = −0.48, *p* = 0.03). Finally, a negative relationship was identified during the SWM task between log RMSSD and the use of strategy (r = −0.46, *p* = 0.02).

##### Log LF/HF Ratio

The high log LF/HF (Table 11) group identified a negative correlation during the SWM task between log RMSSD and strategy (r = −0.45, *p* = 0.02).

The low log LF/HF (Table 11) group identified a positive correlation during the AST between log TP and errors when the “side” cue was given (r = 0.43, *p* = 0.03). A negative association was also found during the AST between log LF and reaction times on incongruent trials (r = −0.68, *p* < 0.001).

## 4. Discussion

In this study, we identified comparisons and differences in neurocognitive performance between high-HRV and low-HRV groups of blue- and white-collar workers. Based on previous literature, it was hypothesized that differences would be observed in neurocognitive performance between high-HRV groups and low-HRV groups; however, we also aimed to explore the specific interactions between HRV and neurocognitive performance within the blue- and white-collar worker population. We define blue-collar occupations as those that involve manual labor with much higher physical demands than white-collar occupations, which we define as managerial or administrative-based work.

Given the strong neurophysiological support and high correlation between RMSSD and the spectral correlate, high frequency (HF), the high-HRV and low-HRV groups were split around the median based on these parameters [12,28,29,30]. Despite contentions of its physiological origins, the sympathovagal balance (LF/HF) was also split around the median, as some research has indicated its significance in determining the activities of the autonomic nervous system [30,31].

### 4.1. White-Collar Differences

The high RMSSD group showed better performance on the tasks, particularly in making fewer errors during the attention-switching task (AST) and the spatial span (SSP) task. This is strongly supported by literature, which indicates that higher parasympathetic activity and increased vagal control (indexed by RMSSD) are associated with better performance [32]. It was observed that higher-HRV participants demonstrated an enhanced ability to stop and change responses during executive function load [33], further supporting the notion that more vagal control of cardiac autonomic activity is associated with superior performance [34]. In contrast to that of the literature, the present study found that the higher-HF (HRV) group made more errors where, typically, it is seen that higher parasympathetic activity is associated with better performance [12]. This may, consequently, highlight the characteristics of the task at hand, playing a major role in moderating the relationship between HRV parameters and neurocognitive performance.

It is widely understood that higher parasympathetic activity, indexed by HRV parameters (RMSSD and HF), is associated with better performance, and conversely, lower HRV parameters (RMSSD and HF) are associated with worse performance [16]. Additionally, it seems that both increased sympathetic activation and decreased parasympathetic activity are the main drivers of this interaction [34]. Notwithstanding, the current findings may suggest that the classic assumptions of higher cardiac vagal control being associated with improved executive function do not universally hold [13]. Instead, it may be task-dependent where, for example, the introduction of a time pressure may require additional cardiovascular adjustment to cope with the increased metabolic needs [13].

### 4.2. White-Collar Correlations

The high-HRV (RMSSD) group showed a positive relationship between RMSSD and the number of errors and a negative relationship between LF/HF HRV and reaction time during the AST. This association between increased vagal tone and more errors contrasts the literature but, as previously stated, may further highlight the importance of task-dependent influences on cardiac autonomic functions [13]. Moreover, supporting much of the literature, the increased parasympathetic dominance (whereby the HF component of the LF/HF variable increased) was associated with faster reaction times [12]. The high RMSSD group also showed numerous associations in the rapid visual processing (RVP) task. In a task of sustained attention (RVP), higher SDNN, LF, HF, and TP were all associated with longer reaction times. The general increase across multiple HRV indices is consistent with research regarding executive stimulation and load, which indicates that as executive function load increases, autonomic HRV input also increases [35].

When split based on HF HRV, the high HF HRV white-collar workers showed an association between high parasympathetic activity (HF HRV) and reduced number of errors on the AST. Again, this is quite consistent across the literature, indicating that increased vagal control relates to better performance [12]. Additionally, the high-HF HRV group observed a relationship between increased sympathetic dominance (indicated by increased LF within the LF/HF HRV parameter) and quicker reaction times during the AST. This may be due to increased mental stress and effort to perform well on the given task, which in turn increases sympathetic dominance [36]. Moreover, during the spatial working memory (SWM) task, increased sympathetic activity was positively associated with errors, and increased vagal control was inversely associated with errors made, again in support of previous literature [34].

Interestingly, the low-RMSSD group showed an inverse association between HRV measures (SDNN, HF, and TP) and reaction time. This implies that as these variables increased, the white-collar workers had faster reaction times. This is also consistent with the literature, which indicates higher vagal tone is associated with better performance [34]. However, an idea presented by Porges [37] suggests that stronger autonomic reactivity (change between baseline and active) is found in individuals with higher resting cardiac vagal tone. Therefore, one may assume that this higher resting vagal tone allows for a stronger stress-induced change in performance [13]. Moreover, these results may further suggest that varying baseline HRV parameters may make an individual susceptible to reacting differently to certain stimuli and causing differing changes in cardiac autonomic input (i.e., someone with a higher baseline HRV may react differently than someone with a lower baseline HRV) [37].

During the AST, the white-collar workers who were classified into the low-HF HRV group showed a positive association between overall HRV (SDNN HRV) and the number of correct responses. The present study also found an inverse relationship between parasympathetic cardiac activity (indexed by HF and RMSSD) and errors, as well as reaction time. These results support previous findings that further highlight the critical role that vagal modulation plays in executive function [38]. Total power (TP) HRV was also found to be inversely correlated to the total number of incorrect responses. Moreover, during the spatial span (SSP) task, increased cardiac vagal input (RMSSD HRV) was correlated to reduced errors made, as consistently proven in the literature [12,16,38,39].

### 4.3. Blue Collar Differences

Differences between high and low HRV for blue-collar workers showed somewhat contradictory results to that of previous literature [12]. Vagally mediated HRV is indexed by both RMSSD and HF HRV. These two HRV variables are observed to be highly correlated [40]. However, the current observations show some differing findings between HF and RMSSD HRV within the blue-collar workers. The blue-collar workers with high RMSSD indicated worse performance with a distinct difference in the ability to detect sequences during the RVP task. This worse performance is contrasted by the high-HF HRV group, which showed fewer errors made as compared to the low-HF HRV group during the SSP task. Both HF HRV and RMSSD represent parasympathetic modulation, and as stated, both are correlated to one another. This unexpected relationship between RMSSD and HF HRV, or lack thereof, was also found by Ravé et al. [41]. This may suggest that HF HRV might reflect more of total HRV (SDNN and TP HRV), whilst RMSSD may be a better true marker of parasympathetic influence in blue-collar workers. Moreover, it is acknowledged that the HRV parameters observed may differ from previous literature due to the variation in sample types studied, which may influence the variance observed.

### 4.4. Blue-Collar Correlations

The blue-collar workforce showed various associations with HRV parameters, and both expected and unexpected results were observed. Higher HRV showed a positive correlation between vagal control (HF HRV) and the longest sequence remembered during the SSP task. This is expected given the results of literature showing that higher vagal activity is associated with better performance [34]. What was unexpected, however, is the inverse relationship found between RMSSD HRV (parasympathetic activity) and the longest sequence remembered. Given that the blue-collar workforce often engages in activities involving their spatial awareness, this finding suggests an element of memory, adding to mental workload, which may have influenced these workers. Further to this notion, it was found that during the spatial working memory (SWM) task, HF HRV was positively correlated to the number of errors made. This reinforces the idea that though the spatial awareness of blue-collar workers may be enhanced, the working memory element may be causing increased mental workload and, therefore, hindering performance.

The low-RMSSD and low-HF group also indicated worse performance, particularly when it comes to a test of sustained attention. During the rapid visual processing (RVP) task, links between low RMSSD and low HF HRV were found with increased errors made, slower reaction times, and fewer sequences being detected were found. This is in support of literature that suggests that lower parasympathetic activity may predispose someone to worse performance [12,34]. Comparatively, the literature indicates that higher resting HRV is associated with better situational awareness, HRV decreases as mental effort increases, and a greater sympathetic dominance negatively affects reaction times and performance [42,43,44].

## 5. Conclusions

The present findings of the blue- and white-collar workers identified many varying differences and associations. However, overall observations regarding cardiac vagal influence seem consistent with the literature, whereby higher RMSSD and HF were linked to better performance [34]. In general, those with higher parasympathetic HRV (HF, RMSSD, and higher LF/HF) showed significantly better performance, while the inverse was observed in those with lower parasympathetic HRV. Interestingly, the blue- and white-collar groups exhibited different correlations and, in some cases, showed the inverse relationship between the same variables. A major notion discerned in the present study is that perhaps increased parasympathetic activity (or vagal modulation) may not simply signify better performance. Rather, it challenges this previous assumption such that the influences on individual HRV parameters may be task-dependent, especially when highly correlated variables (HF HRV and RMSSD) show opposing associations. The differences presented here, therefore, demonstrate the importance of assessing task-dependent HRV parameters. It may not be sufficient to conclude the effect of vagal control on performance in its entirety, particularly when many factors like neurophysiology [45,46], diseased states [47,48,49], and metabolic demands [13], among others, affect HRV and neurocognitive performance. Given these findings, future studies may want to explore additional data around the exact type of work performed in addition to the time of day worked.

## Figures and Tables

**Table 1:** Median for HRV split in white-collar group.

Variable	Median	No. in High Group	No. in Low Group
Log RMSSD (ms)	6.69	n = 20 (41.7%)	n = 28 (58.3%)
Log HF (ms^2^)	5.07	n = 24 (50%)	n = 24 (50%)
Log LF/HF	1.49	n = 24 (50%)	n = 24 (50%)

Table 1 reports the median split values for the white-collar group and the number of participants that were placed in the high and low groups, respectively. Key: HRV = heart rate variability; HF = high frequency; LF/HF = low-frequency to high-frequency ratio; n = sample size; No. = number; RMSSD = root mean square of successive differences.

**Table 2:** Mann–Whitney U test comparing neurocognitive performance between high and low HRV in the white-collar group (n = 48).

HRV Split	Variable	U	Z	*p*	High HRV Mean ± SD	Low HRV Mean ± SD	Mean Difference (High HRV–Low HRV)
Log RMSSD M = 6.69 (ms)	Errors (not next in sequence)	173	−2.43	0.02	1.08 ± 1.2	2.08 ± 1.44	−1
Log HF M = 5.07 ms^2^	Errors (side)	183	−2.18	0.03	5.17 ± 2.44	3.5 ± 2.35	1.67

Table 2 shows the results of the Mann–Whitney U tests performed to compare the neurocognitive performance scores between high and low HRV in the white-collar group. The HRV parameters split that show significance (*p* < 0.05) were log RMSSD, log HF. Key: HRV = heart rate variability; HF = high frequency; M = median; ms = milliseconds; ms^2^ = milliseconds squared; n = sample size; *p* = level of significance (*p* < 0.05); RMSSD = root mean square of successive differences; SD = standard deviation; U = U test statistic; Z = Z score.

**Table 3:** Spearman’s correlation between HRV and neurocognitive performance measures in the high and low log RMSSD groups (white collar).

Log RMSSD Median = 6.69 (ms)	Task	Dependent Variable	Independent Variable	r	*p*
High n = 20	AST	Log RMSSD	Total Incorrect	0.41	0.049
		Log LF/HF	Reaction Time (correct)	−0.42	0.04
	RVP	Log SDNN	Reaction Time (correct)	0.6	0.002
		Log LF	Reaction Time (correct)	0.8	0.005
		Log HF	Reaction Time (correct)	0.9	0.01
		Log TP	Reaction Time (correct)	0.54	<0.001
Low n = 28	RVP	Log SDNN	Reaction Time (correct)	−0.43	0.04
		Log TP	Reaction Time (correct)	−0.46	0.02
	SWM	Log HF	Errors (4 boxes)	−0.41	0.045

Table 3 shows the results from Spearman’s correlation performed between the high-HRV and low-HRV groups, based on log RMSSD, and neurocognitive performance measures on the high and low groups for the white-collar workers. Key: AST = attention-switching task; HF = high frequency; HRV = heart rate variability; LF/HF; low frequency divided by high frequency; ms = milliseconds; n = sample size; *p* = level of significance; r correlation coefficient; RMSSD = root mean square of successive differences; RVP = rapid visual processing task; SDNN = standard deviation of all NN intervals (square root of variance); SSP = spatial span task; TP = total power; < = less than.

**Table 4:** Spearman’s correlation between HRV and neurocognitive performance measures in high and low log HF groups (white collar).

Log HF Median = 5.07 (ms^2^)	Task	Dependent Variable	Independent Variable	r	*p*
High n = 24	AST	Log SDNN	Errors (congruent)	0.41	0.049
		Log LF/HF	Reaction Time (correct)	−0.63	0.001
	SWM	Log HF	Errors (6 boxes)	−0.47	0.02
		Log LF/HF	Errors (6 boxes)	0.47	0.02
Low n = 24	AST	Log SDNN	Total Correct	0.43	0.04
		Log LF	Total Incorrect	−0.44	0.03
		Log HF	Congruent Errors	−0.41	0.046
			Reaction Time (direction)	−0.41	0.049
		Log TP	Total Incorrect	−0.46	0.03
	SSP	Log RMSSD	Errors (not in sequence)	−0.42	0.04

Table 4 shows the results from Spearman’s correlation performed between HRV (log HF) and neurocognitive performance measures in the high and low groups for white-collar workers. Key: AST = attention-switching Task; HF = high frequency; HRV = heart rate variability; LF = low frequency; LF/HF = low frequency divided by high frequency; n = sample size; *p* = level of significance; r correlation coefficient; RMSSD = root mean square of successive differences; SDNN = standard deviation of all NN intervals (square root of variance); SSP = spatial span task; SWM = spatial working memory task; TP = total power.

**Table 5:** Spearman’s correlation between HRV and neurocognitive performance measures in high and low log LF/HF groups (white collar).

Log LF/HF Median = 1.49	Task	Dependent Variable	Independent Variable	r	*p*
High n = 24	SSP	Log RMSSD	Errors (not next in sequence)	−0.48	0.02
Low n = 24	AST	Log LF	Reaction Time (direction)	0.49	0.01
		Log LF/HF *	Reaction Time (correct) *	−0.52	0.009
		Log TP	Reaction Time (direction)	0.41	0.049
	RVP	Log SDNN	Reaction Time (correct)	0.42	0.04
		Log HF	Reaction Time (correct)	0.51	0.01
		Log LF/HF *	Reaction Time (correct) *	−0.77	<0.001
	SWM	Log HF	Errors (6 boxes)	−0.55	0.005
		Log LF/HF *	Errors 6 (boxes) *	0.57	0.003
		Log TP	Errors 6 (boxes)	−0.44	0.03

Table 5 shows the results from Spearman’s correlation performed between HRV (log LF/HF) and neurocognitive performance measures on the high and low groups for white-collar workers. Key: AST = attention-switching task; HF = high frequency; HRV = heart rate variability; LF = low frequency; LF/HF = low frequency divided by high frequency; n = sample size; *p* = level of significance; r correlation coefficient; RMSSD = root mean square of successive differences; RVP = rapid visual processing; SDNN = standard deviation of all NN intervals (square root of variance); SSP = spatial span task; SWM = spatial working memory task; TP = total power; * = variables used in multiple regression analysis; < = less than.

**Table 6:** Multiple regression between log LF/HF (white-collar low-HRV group) and neurocognitive performance measures (n = 24).

Low White Collar (n = 24)	R	R Square	Adjusted R Square	Std. Error of the Estimate	*p*
Log LF/HF	0.71	0.51	0.43	0.64	0.002
	B	Std. Error	Beta	t	*p*
(Constant)	4.13	1.24		3.34	0.003
Reaction Time (AST)	0.00	0.001	0.03	0.13	0.90
Reaction Time (RVP)	−0.009	0.003	−0.57	−2.94	0.008
Errors 6 (SWM)	0.11	0.08	0.28	1.45	0.16

Table 6 displays the multiple regression performed between the log LF/HF of the low group for white-collar workers with performance measures on the neurocognitive tasks: reaction time on correct trials (AST), reaction time for correct sequences (RVP), and errors made on the six-box trial (SWM). Key: AST: attention-switching task; B = unstandardized regression coefficient; LF/HF = low frequency divided by high frequency; n = sample size; *p* = level of significance; R = correlation coefficient; RVP = rapid visual processing; R square = proportion of variance; SWM = spatial working memory; Std. = standard; t = t statistic.

**Table 7:** Median for HRV split in blue-collar group.

Variable	Median	No. in High Group	No. in Low Group
Log RMSSD (ms)	6.69	n = 24 (45.3%)	n = 29 (54.7%)
Log HF (ms^2^)	4.62	n = 26 (49.1%)	n = 27 (50.9%)
Log LF/HF	1.83	n = 25 (47.2%)	n = 28 (52.8%)

Table 7 reports the median split values for the blue-collar group and the number of participants that were placed in the high or low group, respectively. Key: HRV = heart rate variability; HF = high frequency; LF/HF = low-frequency to high-frequency ratio; n = sample size; No. = number; RMSSD = root mean square of successive differences.

**Table 8:** Mann–Whitney U test comparing neurocognitive performance between high and low HRV in the blue-collar group (n = 53).

HRV	Variable (Unit)	U	Z	*p*	High HRV Mean ± SD	Low HRV Mean ± SD	Mean Difference (High-Low)
Log RMSSD M = 6.69 (ms)	Signal Detection	234	−2.07	0.04	0.8 ± 0.08	0.9 ± 0.07	−0.04
	Sequences Detected	238	−1.99	0.047	33.08 ± 12.26	40.61 ± 9.79	−7.53
	Sequences Missed	238	−1.99	0.047	20.92 ± 12.26	13.39 ± 9.79	7.53
Log HF M = 4.62 (ms^2^)	Total Errors (not next in sequence)	238	−2.0	0.046	8 ± 5.95	11.29 ± 6.56	−3.29
Log LF/HF M = 1.83	Sequences Detected	225	−2.24	0.03	41.19 ± 9.62	33.07 ± 12.03	8.12
	Sequences Missed	225	−2.24	0.03	12.81 ± 9.62	20.92 ± 12.03	−8.12

Table 8 shows the results of Mann–Whitney U tests performed on the high and low-HRV groups for blue-collar workers. Median split was performed between RMSSD, HF, and LF/HF, as well as their log-transformed counterparts. The parameters that showed significant differences (*p* < 0.05) were RMSSD, log RMSSD, HF, log HF, LF/HF, and log LF/HF. Key: HF = high frequency; HRV = heart rate variability; LF/HF = low frequency divided by high frequency; M = median; ms = milliseconds; ms^2^ = milliseconds squared; n = sample size; *p* = level of significance; RMSSD = root mean square of successive differences; SD = standard deviation; U = U test statistic; Z = Z score.

**Table 9:** Spearman’s correlation between HRV and neurocognitive performance measures in high and low log RMSSD groups (blue collar).

Log RMSSD Median = 6.69 (ms)	Task	Dependent Variable	Independent Variable	r	*p*
High n = 24	SSP	Log RMSSD	Longest sequence	−0.4	0.04
		Log SDNN	Longest sequence	0.49	0.001
			Errors (not in sequence)	−0.38	0.049
		Log LF/HF	Longest sequence	−0.51	0.01
			Errors (not in sequence)	0.39	0.04
	SWM	Log HF	Longest sequence	0.57	0.002
Low n = 29	RVP	Log RMSSD	Errors (not next in sequence)	0.44	0.03
	SSP	Log SDNN	Errors (not next in sequence)	0.44	0.03
		Log TP	Reaction time (correct)	0.43	0.03
			Longest sequence	−0.4	0.045

Table 9 shows the results from Spearman’s correlation performed between HRV (log RMSSD) and neurocognitive performance measures for the high and low groups of blue-collar workers. Key: AST = attention-switching Task; HF = high frequency; HRV = heart rate variability; LF/HF; low frequency divided by high frequency; ms = milliseconds; n = sample size; *p* = level of significance; r correlation coefficient; RMSSD = root mean square of successive differences; RVP = rapid visual processing task; SDNN = standard deviation of all NN intervals (square root of variance); SSP = spatial span task; SWM = spatial working memory task; TP = total power.

**Table 10:** Spearman’s correlation between HRV and neurocognitive performance measures in high and low log HF groups (blue collar).

Log HF Median = 4.62 (ms^2^)	Task	Dependent Variable	Independent Variable	r	*p*
High n = 26	RVP	Log SDNN	Reaction Time (correct)	0.54	0.003
		Log HF	Reaction Time (correct)	0.41	0.03
		Log TP	Reaction Time (correct)	0.48	0.01
Low n = 27	AST	Log SDNN	Total Incorrect	0.54	0.01
		Log HF	Total Incorrect	0.41	0.04
	RVP	Log LF/HF	Total Sequences Detected	−0.45	0.02
			Total Sequences Missed	0.45	0.02
	SSP	Log SDNN	Errors (not in sequence)	−0.48	0.03
	SWM	Log RMSSD	Strategy	−0.46	0.02

Table 10 shows the results from Spearman’s correlation performed between HRV (log HF) and neurocognitive performance measures on the high and low groups of blue-collar workers. Key: AST = attention-switching task; HF = high frequency; HRV = heart rate variability; LF/HF; low frequency divided by high frequency; n = sample size; p = level of significance; r correlation coefficient; RMSSD = root mean square of successive differences; RVP = rapid visual processing task; SDNN = standard deviation of all NN intervals (square root of variance); SSP = spatial span task; SWM = spatial working memory task; TP = total power.

**Table 11:** Spearman’s correlation between HRV and neurocognitive performance measures in high and low log LF/HF groups (blue collar).

Log LF/HF Median = 1.83	Task	Dependent Variable	Independent Variable	r	*p*
High n = 25	SWM	Log RMSSD	Strategy	−0.45	0.02
Low n = 28	AST	Log LF	Reaction Time (incongruent)	−0.68	<0.001
		Log TP	Errors (side)	0.43	0.03

Table 11 shows the results from Spearman’s correlation performed between HRV (log LF/HF) and neurocognitive performance measures on the high and low groups of blue-collar workers. Key: AST = attention-switching task; HRV = heart rate variability; LF/HF; low frequency divided by high frequency; n = sample size; *p* = level of significance; r correlation coefficient; RMSSD = root mean square of successive differences; SWM = spatial working memory task; TP = total power; < = less than.

## Data Availability

All data relevant to the present study are available from the corresponding author upon reasonable request.
